# The pathways of pluripotent stem cells to clinical applications

**DOI:** 10.1186/s41232-024-00317-6

**Published:** 2024-01-15

**Authors:** Jun K. Yamashita

**Affiliations:** Department of Cellular and Tissue Communications, Graduate School of Medicine, the University of Tokyo, Tokyo, 113-8655 Japan

More than 15 years have passed since the groundbreaking establishment of induced pluripotent stem (iPS) cells, and we are now witnessing the fruition of iPS cell research in the form of novel therapies reaching clinical application, a goal envisioned from the inception of this field. In this thematic series, titled “Pluripotent Stem Cell Research Reaching Clinical Applications,” we are pleased to introduce two comprehensive reviews on new iPS cell therapies that have achieved clinical application.

The first review delves into iPS cell-derived neural precursor cell transplantation therapy for Parkinson’s disease. Morizane and colleagues performed pioneering work in iPS cell-based cell therapy. Being at the forefront necessitated navigating uncharted territories to establish the clinical application pathway. Critical questions such as the identification of necessary cells, their induction and preparation methods, quality control processes, transplantation strategies including cell quantity and site, the extent of immunosuppression required, and the evaluation and management of therapeutic effects and adverse effects all had to be meticulously addressed. The journey of setting these standards and successfully reaching clinical application is indeed commendable, and this review offers a glimpse into its essence.

The second review presents a shift from the traditional focus of pluripotent stem cell therapy on regenerative medicine to targeting cancer treatment. The group led by Aoki, Motohashi, and Koseki has endeavored to replicate the anticancer effects of invariant natural killer T (iNKT) cells, previously observed in knockout mouse experiments, by inducing iNKT cells from iPS cells. They demonstrated anticancer effects of these cells in vivo using mouse iPS cells and, based on this proof of concept, have developed techniques for inducing and expanding iNKT cells from human iPS cells, culminating in their administration to humans. While immune responses are complex and may vary between mice and humans, it is hoped that the steady progress of research will be replicated in human applications.

The clinical application of pluripotent stem cells is beginning to broaden its horizons beyond traditional regenerative medicine, with the recent initiation of applications in cancer treatment. Looking forward, it is anticipated that the scope of these applications will expand further, targeting various diseases and pathological conditions, potentially including antiaging and rejuvenation.

I would like to extend my deepest gratitude to the distinguished researchers who have made invaluable contributions to this special issue. It is my earnest hope that these insightful reviews will serve as a catalyst for future research endeavors in the fields of inflammation and regeneration, inspiring and guiding our esteemed readers.

